# High titer MVA and influenza A virus production using a hybrid fed-batch/perfusion strategy with an ATF system

**DOI:** 10.1007/s00253-019-09694-2

**Published:** 2019-02-23

**Authors:** Daniel Vázquez-Ramírez, Ingo Jordan, Volker Sandig, Yvonne Genzel, Udo Reichl

**Affiliations:** 1 Max Planck Institute for Dynamics of Complex Technical Systems, Sandtorstr. 1, 39106 Magdeburg, Germany; 2 ProBioGen AG, Goethestr. 54, 13086 Berlin, Germany; 3 Chair for Bioprocess Engineering, Otto-von-Guericke-University Magdeburg, Universitätsplatz 2, 39106 Magdeburg, Germany

**Keywords:** Viral vaccine production, Process intensification, On-line monitoring

## Abstract

A cultivation strategy to increase the productivity of Modified Vaccinia Ankara (MVA) virus in high-cell density processes is presented. Based on an approach developed in shake flask cultures, this strategy was established in benchtop bioreactors, comprising the growth of suspension AGE1.CR.pIX cells to high cell densities in a chemically defined medium before infection with the MVA-CR19 virus strain. First, a perfusion regime was established to optimize the cell growth phase. Second, a fed-batch regime was chosen for the initial infection phase to facilitate virus uptake and cell-to-cell spreading. Afterwards, a switch to perfusion enabled the continuous supply of nutrients for the late stages of virus propagation. With maximum infectious titers of 1.0 × 10^10^ IU/mL, this hybrid fed-batch/perfusion strategy increased product titers by almost one order of magnitude compared to conventional batch cultivations. Finally, this strategy was also applied to the production of influenza A/PR/8/34 (H1N1) virus considered for manufacturing of inactivated vaccines. Using the same culture system, a total number of 3.8 × 10^10^ virions/mL was achieved. Overall, comparable or even higher cell-specific virus yields and volumetric productivities were obtained using the same cultivation systems as for the conventional batch cultivations. In addition, most viral particles were found in the culture supernatant, which can simplify further downstream operations, in particular for MVA viruses. Considering the current availability of well-described perfusion/cell retention technologies, the present strategy may contribute to the development of new approaches for viral vaccine production.

**Electronic supplementary material:**

The online version of this article (10.1007/s00253-019-09694-2) contains supplementary material, which is available to authorized users.

## Introduction

Modified Vaccinia Ankara (MVA) virus is a highly attenuated poxvirus with promising properties as a vectored vaccine. MVA initiates but cannot complete a full replication cycle in human recipients and is therefore immunogenic similar to live virus vaccines (Gomez et al. 2013) but with safety properties resembling inactivated virus vaccines (Gilbert et al. 2006; Cebere et al. 2006; Webster et al. 2005; Stickl et al. 1974; Mayr 2003). MVA recombinants expressing different viral heterologous antigens have been tested in pre-clinical and clinical trials as candidate vaccines against infectious diseases such as AIDS, influenza, severe acute respiratory syndrome (SARS), and human respiratory syncytial virus (RSV) infection (Boukhebza et al. 2012; Gilbert 2013; Gomez et al. 2011, 2012). Conventionally, MVA seed virus stocks considered for vaccine manufacturing are produced in chicken embryo fibroblast (CEF) that are fully permissive for MVA. Using well-established protocols (Altenburg et al. 2014; Cotter et al. 2017), large-scale production of MVA recombinants for immunization campaigns would also have to rely on propagation in CEF (Altenburg et al. 2014). However, because supply with the primary cell cultures can be challenging for large scale manufacturing, production of MVA recombinants is being investigated for continuous suspension cell lines, such as the duck cell lines AGE1.CR and AGE1.CR.pIX (Jordan et al. 2009; Lohr et al. 2009), or the duck embryonic stem cell-derived EB66 cells (Léon et al. 2016).

Influenza viruses are usually processed to provide inactivated vaccines against seasonal epidemics (Soema et al. 2015). Production of influenza vaccines in the past 70 years relied on embryonated chicken eggs. Disadvantages associated with this substrate are the use of an animal-derived substrate and the potential shortage of eggs especially in case of a pandemic emergency. Recently, two cell culture-based vaccines produced either in MDCK cells or in insect cells using the baculovirus expression vector system were approved by the FDA (Buckland 2015). In addition, efficient production of influenza virus has also been shown for other suspension cell lines including Vero cells (Litwin 1992; Paillet et al. 2009), PER.C6 cells (Pau et al. 2001), HEK293 cells (Le Ru et al. 2010), EB66 cells (Brown and Mehtali 2010; White et al. 2018), and AGE1.CR as well as AGE1.CR.pIX cells (Jordan et al. 2013; Lohr et al. 2012; Lohr 2014).

Cell culture-derived viral vaccines are typically produced in biphasic processes. They comprise an initial cell growth phase and a virus replication phase that initiates with inoculation by seed virus. After the viral genome is amplified and viral proteins are produced, virions are assembled and progeny virus particles released (Aunins 2000). Typically, cells are first cultivated in batch mode and infected in the late exponential growth phase at concentrations in the order of 10^5^–10^6^ cells/mL, with or without a partial exchange of culture medium (Aunins 2000; Tapia et al. 2016). Titers of wild-type MVA obtained in adherent cultures of CEF cells in serum-containing medium are in the range of 10^7^–10^9^ infectious units (IU) per mL (Gilbert et al. 2005; Meiser et al. 2003). The avian cell lines AGE1.CR.pIX (Jordan et al. 2009; Lohr et al. 2009; Lohr 2014), EB14 (Guehenneux and Pain 2005) and EB66 (Léon et al. 2016) also yield titers in the order of 10^8^ IU/mL but with the advantage of enabling suspension cell culture processes. However, one caveat is that these processes require induction of suspended cell aggregates for efficient replication of MVA. A novel MVA derivative, MVA-CR19, was adapted to propagation in true single-cell suspension cultures without the requirement for addition of medium to induce cell aggregation (Jordan et al. 2013).

Scalable and intensified processes that yield high titers are desirable to secure adequate supply with vaccines. In the case of MVA, high virus titers are required because MVA does not replicate in human recipients and therefore is not amplified at the site of injection. Concentrated doses of 10^8^–10^9^ IU/mL are estimated to be required for clinical applications of MVA (Gomez et al. 2013; Altenburg et al. 2014). In the case of influenza vaccines, intensification is desirable because composition of multivalent vaccines changes every year, and the time is short between selection of seasonal virus strains and desired start of vaccination.

Increasing the concentration of host cells is a standard approach to intensify production processes for biologicals. Processes at high cell density (HCD) allow the use of compact bioreactors with high volumetric production rates and can be adjusted to viable cell densities of 10^7^–10^8^ per mL (Clincke et al. 2013). Such cell densities can be achieved in perfusion mode, which allows for a continuous addition of fresh medium and removal of toxic by-products (such as lactate and ammonium) while retaining cells in the bioreactor using different retention systems. The production of recombinant proteins in perfusion is typically performed at high-medium exchange rates of 1–3 media volumes per reactor volume per day (1/day) or 0.05–0.5 nL/(cell × day) (Konstantinov et al. 2006). To maintain cultures in a proliferative state at constant high cell densities, a controlled and continuous removal of cells from the bioreactor is performed, the so-called “cell bleed” (Clincke et al. 2013; Deschênes et al. 2006; Hiller et al. 1993)**.** Novel cultivation strategies such as n-1 perfusion/high-seed fed-batch (Yang et al. 2014), concentrated fed-batch (FB) (Yang et al. 2016), and hybrid perfusion FB (Hiller et al. 2017) have been developed to minimize media use while maintaining cell-specific and volumetric productivities as well as guaranteeing consistent product quality.

In the field of viral vaccine production, high cell concentrations up to 5 × 10^7^ cells/mL were used for the propagation of influenza A/PR/8/34 (H1N1) virus (Genzel et al. 2014) and the MVA-CR19 virus strain (Vazquez-Ramirez et al. 2018). At those cell concentrations, virus propagation must be performed at optimal pH, temperature, and nutrient concentrations to avoid the so-called “cell density effect”, a reduction in cell-specific virus yield often observed for concentrations exceeding 5 × 10^6^ cells/mL (Lindsay and Betenbaugh 1992; Maranga et al. 2003).

Several improvements were reported for the HCD production of influenza A/PR/8/34 (H1N1) virus. Cultivations were performed in perfusion cultures for both cell and virus propagation phases using an acoustic filter (Petiot et al. 2011) or an alternating tangential flow (ATF) system for cell retention (Genzel et al. 2014). The perfusion systems differed in the partitioning of the virus. Whereas the acoustic filter allowed a continuous virus harvest, ATF systems with membrane pores sizes < 0.5 μm retained most released viral particles in the bioreactor. In addition, the continuous permeate flow through the hollow fiber membrane resulted in product losses due to unspecific binding or membrane fouling by entrapment of cellular debris and virus particles within the membrane pores (Genzel et al. 2014). Although cell-specific virus yields comparable to conventional batch processes were obtained for both cell retention systems, the volumetric productivity (the amount of virus per volume of medium spent and time) was lower (Genzel et al. 2014). This strategy would therefore be less competitive because of the increased cost of goods (COGs) for its implementation in large-scale (Pollock et al. 2013). In an attempt to produce MVA virus (MVA-CR19 strain) at high concentrations in AGE1.CR.pIX cells (5 × 10^7^ cells/mL), perfusion using a medium free of animal-derived components was implemented (Vazquez-Ramirez et al. 2018). This method resulted in virus retention and yielded lower cell-specific and volumetric productivities compared to conventional cell density cultivations. A detailed analysis of several cultivation strategies in shake flasks demonstrated that a FB phase followed by a daily medium exchange of 90% can result in an improvement of both cell-specific yield and volumetric productivity, even surpassing conventional cell density processes performed as a control (Vazquez-Ramirez et al. 2018). While shake flask experiments are essential for the characterization of production processes, they are insufficient for establishing strategies for large-scale manufacturing of vaccines.

Hence, in the present study, an optimized HCD process was developed in a controlled and scalable cultivation system to provide the required high yields of MVA virus. HCD cultures were achieved in a 1 L bioreactor with an ATF perfusion system using a manual perfusion control. Perfusion rates were adjusted applying a fixed cell-specific perfusion rate (CSPR), which is the volume of medium provided to a single cell per day (Ozturk 1996). During virus production, the cells were cultivated in FB followed by a continuous medium exchange with the same ATF perfusion system used for the cell proliferation phase (hybrid FB/perfusion strategy). Yields and productivity of this process confirmed shake flask results. To investigate options for the use of this strategy to other viral vaccine production processes, propagation of influenza A virus was also tested. Again, a similar or even higher productivity compared to conventional cell density cultivations was obtained.

## Materials and methods

### Cells and medium

The AGE1.CR.pIX cell line (here named CR.pIX) is directly derived from the avian cell line AGE1.CR, which was generated from Muscovy duck retina cells (Jordan et al. 2009). The CR.pIX cells differ from their progenitor AGE1.CR in that they express the pIX protein of human adenovirus (Jordan et al. 2009), a virus that is not related to MVA or influenza viruses. Suspension CR.pIX cells were cultivated in chemically defined CD-U3 medium (Biochrom GmbH) with a glucose concentration of 33–40 mM, supplemented with glutamine (Sigma, Lot SLBS8600) and alanine (Sigma, Lot BCBS2461V) to a final concentration of 2 mM. In addition, recombinant insulin-like growth factor (LONG-R3IGF, Sigma, Lot LOS6008) was added at 10 ng/mL final concentration. Cells were passaged every 3–4 days at a seed concentration of 0.8 × 10^6^ cells/mL.

### Bioreactor cultivations

CR.pIX cells were inoculated in a 1 L (nominal volume) benchtop bioreactor (BIOSTAT®B plus, Sartorius AG) at 0.8 × 10^6^ cells/mL in a working volume (*V*_w_) of 0.6–0.8 L (Table 1). Bioreactors were operated at 37 °C, pH 7.2, and a stirring speed of 120–160 rpm. Dissolved oxygen concentration (DO) was controlled at 40% by pulsed aeration with pure oxygen through a 20-μm pore size micro-sparger unit to a maximum of 29–38 cm^3^/min. Cells were initially cultivated in batch until a glucose concentration of 14–17 mM (60–72 h after inoculation) was reached. At that point, perfusion was started using an ATF2 perfusion system controlled by the C24U-V2.0 controller from Refine Technology and polysulfone hollow fiber cartridges with pore sizes of 500 kDa (UFP-500-E-4X2MA, GE Healthcare) and 0.65 μm (CFP-6-D-4X2MA, GE Healthcare), or polyethersulfone hollow fiber cartridges of 0.2 μm (S06-P20U-10-S, Spectrum Labs) (Table 1). Cell suspension flow rate within the hollow fiber was set at 1.0 L/min, and defined perfusion rates were applied to achieve cell densities > 25 × 10^6^ cells/mL.

**Table 1:** Summary of process parameters and performance during the propagation of CR.pIX cells for all analyzed cultivations

Cultivation	Working volume [mL]	Hollow fiber module	Initial VCD [10^6^ cells/mL]	*μ*_mean_ [1/h]	*μ*_max_ [1/h]	Actual average CSPR [nL/(cell × day)]	Duration [h]***
MVA-CR19 virus							
Perfusion*	800	500 kDa	0.8	0.019 (*n* = 12, SD = 0.005)	0.026	0.057 (*n* = 24, SD = 0.006)	247
Hybrid 1	600	0.65 μm	1.0	0.021 (*n* = 12, SD = 0.006)	0.029	0.068 (*n* = 18, SD = 0.013)	191
Hybrid 2	600	0.20 μm	8.0	0.024 (*n* = 4, SD = 0.005)	0.031	0.057 (*n* = 7, SD = 0.011)	50
Influenza A virus							
Perfusion	800	500 kDa	1.0	0.017 (*n* = 18, SD = 0.011)	0.055	0.072 (*n* = 24, SD = 0.010)	183
Hybrid	600	0.65 μm	1.0	0.019 (*n* = 7, SD = 0.004)	0.025	0.119 (*n* = 20, SD = 0.061)**	168
Batch	800	NA	0.8	0.020 (*n* = 6, SD = 0.009)	0.033	NA	82

*Vazquez-Ramirez et al. (2018)

**Non-constant CSPR: perfusion rates were automatically adjusted to maintain the pH at a set point of 7.2 and not based on the actual viable cell densities

***Time from cells inoculation to infection

Perfusion flow rates during the cell growth phase were adjusted manually every 12 or 24 h. For that, viable cell densities were measured off-line, and the corresponding flow rates for that sampling time were calculated to assure a CSPR of 0.06 nL/(cell × day), which is the optimal exchange rate for CR.pIX cells based on their glucose consumption rate (Vazquez-Ramirez et al. 2018). Expected viable cell densities and the corresponding perfusion flow rates after 12 or 24 h were calculated taking into account a maximum cell-specific growth rate of *μ* = 0.026 h^−1^ (data not shown). A linear profile between sampling time points was achieved using the cascade control of the BIOSTAT®B plus module. Two hours before infection, one reactor volume was exchanged with fresh medium using the ATF system. A summary of key parameters of all perfusion cultivations is presented in Table 1.

**Fig. 1:**
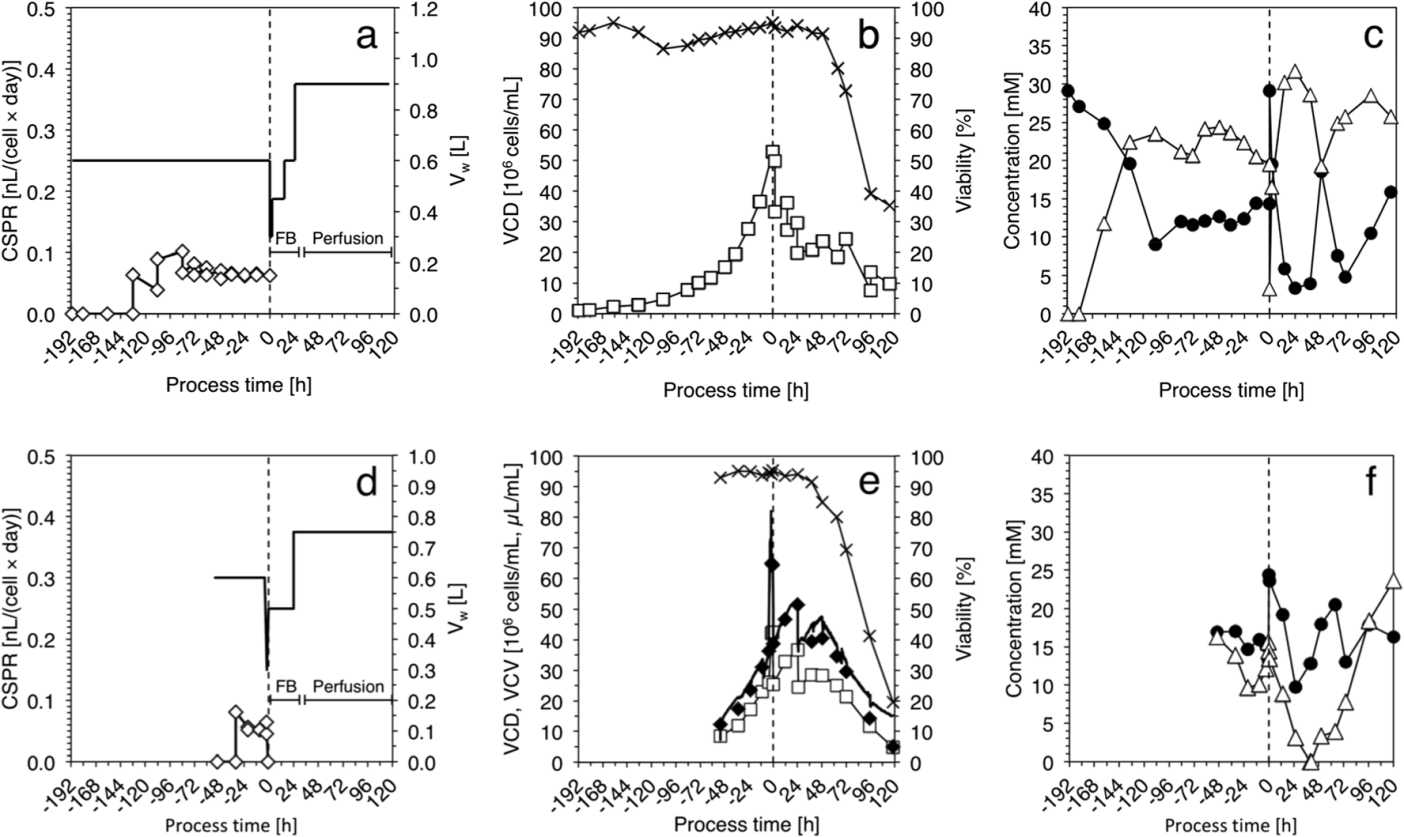
Cultivation parameters and cell growth for two hybrid FB/perfusion variants, Hybrid 1 (**a**–**c**) and Hybrid 2 (**d**–**f**), for the production of MVA-CR19 virus at high cell density. **a**, **d** Cell-specific perfusion rate, CSPR, (empty diamonds) and working volume, *V*_w_, (continuous line). **b**, **e** Viable cell density, VCD (squares); on-line viable cell volume, VCV (continuous line); off-line VCV (full diamonds) (for VCV calculations see “Materials and Methods” section), and viability (crosses). **c**, **f** Glucose (filled circles) and lactate (triangles) concentration. Time of infection, 0 h (vertical dashed line)

After medium exchange, bioreactors were infected either with MVA-CR19 or influenza A virus A/PR/8/34 (H1N1). For MVA-CR19 virus, two bolus feeding regimes followed by a perfusion regime, namely Hybrid 1 (Fig. 1a) and Hybrid 2 (Fig. 1d), were applied to optimize virus propagation at high cell densities. For Hybrid 1, one-half of the cell suspension was discarded, and the virus production phase was started with 0.3 L of cell suspension to allow for a FB with a volume expansion up to threefold higher than its initial *V*_w_, as described before (Vazquez-Ramirez et al. 2018). The FB was started immediately after virus infection with the addition of 0.15 L of fresh medium to obtain the minimum culture volume at which the bioreactor impeller was at least 2 cm below the medium surface. The FB continued with bolus feedings of 0.15 L at 12 h post infection (hpi) and 0.3 L at 24 hpi. Finally, perfusion was performed at a rate of one reactor volume per day 36–120 hpi (Fig. 1a). MVA-CR19 virus particles (200–400 nm nominal size) were harvested using the same ATF hollow fiber module (0.65 μm pore size) as in the cell growth phase.

Similarly, for Hybrid 2, one bolus feed of 0.2 L was done immediately after virus infection followed by a second feed of 0.25 L at 24 hpi. Subsequently, perfusion was again performed at a rate of one reactor volume per day 36–120 hpi (Fig. 1d). In order to improve the virus harvest, the 0.2-μm ATF module used during the cell growth phase was replaced by a new 0.65-μm module at 36 hpi.

For influenza A virus production, a hybrid strategy (Fig. 3a) similar to the one assessed for MVA-CR19 virus was established. Reference processes, one operated completely in perfusion with total virus retention and one in batch at conventional cell densities, were performed as proposed previously by Genzel et al. (2014). A detailed description of the process parameters during cell expansion (Table 1) and performance during virus propagation (Table 2) is presented in the “Results” section.

**Table 2:** Yields for different cultivation strategies during the virus propagation phase

Cultivation	Working volume [mL]^a^	Hollow fiber module	Harvest volume [mL]	Harvest titer^b^	*t*_T_ [day]^c^	Maximum viable cell number [10^9^]	*Y*_v/cell_^d^	P_V_^e^
MVA-CR19 virus								
Perfusion*	800	500 kDa	800	3.2 × 10^9^	13.0	66.4	38	1.0 × 10^10^
FB + daily harvest (F + D)*	60	NA	95	5.3 × 10^9^	10.0	1.9	270	2.6 × 10^11^
Hybrid 1	900	0.65 μm	900	1.0 × 10^10^	10.9	21.9	410	1.3 × 10^11^
Hybrid 2	750	065 μm	750	1.0 × 10^10^	8.2	21.3	352	2.8 × 10^11^
Influenza A virus								
Hybrid	810	0.65 μm	810	3.80 × 10^10^	9.5	23.7	1300	5.43 × 10^11^
Perfusion	800	500 kDa	800	8.05 × 10^9^	9.6	19.0	340	1.81 × 10^11^
Batch	800	NA	800	5.23 × 10^9^	8.0	3.1	1344	6.53 × 10^11^

^a^For FB-based processes: maximum working volume

^b^In IU/mL for MVA virus and total virions/mL(estimated from HA measurements) for influenza A virus

^c^Total time from cell inoculation to maximum titer

^d^Cell-specific virus yield in IU/cell for MVA virus and total virions/cell (from HA) for influenza A virus

^e^Volumetric productivity in IU/(L × day) for MVA virus and virions/(L × day) (from HA) for influenza A virus

*Vazquez-Ramirez et al. (2018)

### Viruses

All infections with MVA-CR19 virus were carried out with the working bank no. 22.08.2013 (4.41 × 10^8^ infectious units per mL (IU/mL)) derived from a virus seed (Jordan et al. 2013) kindly provided by ProBioGen AG. MVA-CR19 virus seed aliquots were treated for 1 min in a sonication water bath to break up virus aggregates, diluted in fresh medium with a volume equal to 5–6% of the bioreactor *V*_w_, and added to the cell culture to an MOI (multiplicity of infection) of 0.05 IU/cell.

For all experiments with MVA-CR19 virus, the infectious titers were determined taking into account its potential application as a (live) viral vector (Jordan et al. 2013). Vaccinia viruses usually replicate in a highly cell-associated fashion. Therefore, for the quantification of total virus titers (intra- and extracellular), the cell suspensions were treated for cell lysis. The lysates were obtained by three freeze/thaw cycles (− 80°C/RT) followed by 1 min in a sonication water bath (45 kHz). Cellular debris was removed by centrifugation at 1500×*g* at room temperature for 10 min. For the quantification of virus released by host cells into supernatant, the samples were centrifuged at 200×*g* at RT for 5 min. The cell-free supernatant was also subjected to three freeze/thaw cycles before storage (Jordan et al. 2013). All virus samples were stored in aliquots of 0.5–1 mL at − 80°C. The number of infectious units was determined as described previously by Jordan et al. (2009) with a relative standard deviation of ± 0.4 log. The resulting titers are expressed as IU/mL.

The studies with human influenza A virus were performed with MDCK-derived virus seed A/PR/8/34 H1N1 (Robert Koch Institute, Amp. 3138) that was adapted to CR.pIX cells after three passages. The infectious titer of the adapted virus seed was determined by a TCID_50_ assay as 1.48 × 10^7^ IU/mL. All bioreactor experiments were performed at an MOI of 1 × 10^−3^ in the presence of 1 × 10^−6^ U trypsin/cell (Gibco, no. 27250–018; prepared in PBS to 500 U/mL) to facilitate progress of infection. As opposed to MVA, the main application for influenza virus preparations is inactivated vaccine where the total concentration of the viral hemagglutinin protein as an antigen is decisive. For this reason, total virus particle concentrations were estimated by a hemagglutination (HA) assay as previously described by Kalbfuss et al. (2008). HA titers, expressed as log HA units per test volume (log HAU/0.1 mL), were converted to virions/mL assuming the binding of one virus particle per erythrocyte and an erythrocyte concentration of 2 × 10^7^ cells/mL, by:

1 $$ {c}_{\mathrm{virus}}=2\times {10}^7\times {10}^{\left(\log\ \mathrm{HAU}/0.1\ \mathrm{mL}\right)} $$

with *c*_virus_ as the total virus concentration in virions/mL. The relative standard deviation of the method was ± 0.09 log (HA units/0.1 μL) (Kalbfuss et al. 2008).

The cell-specific virus yield (*Y*_v/cell_) was calculated based on the total (i.e., intra- and extracellular) number of infectious virus particles (*vir*_T_, infectivity assay) for MVA virus and on the total number of all virus particles (*c*_virus_, HA assay) for influenza A virus, taking into account the total number of viable cells at the time of sampling (cell_T_). The latter differed from the number of viable cells at the time of infection since cell growth was typically observed up to 36–48 hpi.

Similarly, the volumetric productivity (*P*_V_) was calculated considering vir_T_, the total spent medium during cell growth and virus replication phase (*V*_T,_ L), and the total process time (*t*_T,_ day), by:

2 $$ {P}_{\mathrm{V}}={\mathrm{vir}}_{\mathrm{T}}/\left(\ {V}_{\mathrm{T}}\times {t}_{\mathrm{T}}\right) $$

### Determination of cell and metabolite concentrations

Samples of 6–8 mL from bioreactor cultures were taken with a syringe through a Luer-Lock-septum in 12 or 24-h intervals and stored at − 80°C until analysis. A validated assay using a Bioprofile 100 Plus (Nova Biomedical) was used to determine glucose and lactate concentrations as described previously (Lohr et al. 2009). Viable cell density (VCD, cells/mL), cell viability (%), and average cell diameter (μm) were determined with the cell counter Vi-CELL™ XR (Beckman Coulter) using a previously validated measuring program with a relative standard deviation of 2.5% for AGE.CR and CR.pIX cells (Lohr 2014). Cells analyzed from a total of 100 images were clustered in diameter classes in the range of 8.1–29.9 μm for calculation of the total viable cell volume per culture volume (VCV, μL/mL) using the cell number and cell diameter distribution. For some cultivations, an Incyte® capacitance probe connected to an Arc View 265 controller (Hamilton Bonaduz AG) was evaluated for its performance to deliver on-line VCV data. The on-line system was configured to provide the cell culture’s permittivity (*ε*, pF/cm), which correlates directly to the VCV. On-line permittivity was converted to VCV applying a correlation factor VCV/*ε* of 1.8 obtained from previous cultivations (data not shown).

## Results

A strategy previously reported for production of MVA-CR19 virus at high cell densities in shake flasks (Vazquez-Ramirez et al. 2018) was transferred to a controlled stirred tank bioreactor with an ATF2 system for cell retention. The method transfer was investigated for production of MVA and influenza A virus.

### MVA-CR19 virus propagation using hybrid FB/perfusion

For the MVA-CR19 virus, this process was adapted for its implementation in a 0.6-L (*V*_w_) bioreactor. During the cell growth phase of variant “Hybrid 1”, an actual CSPR of 0.068 nL/(cell × day) (Fig. 1a and Table 1) was obtained, which was comparable to the target 0.06 nL/(cell × day). This perfusion rate enabled a cell expansion from 1 to > 50 × 10^6^ cells/mL (Fig. 1b) with a *μ*_mean_ of 0.021 1/h (Table 1). This was comparable to the assumed specific growth rate of 0.026 1/h for perfusion and was within the range of 0.016–0.023 1/h from previous reports for batch cultures (Lohr et al. 2012). Manual control of the CSPR during the cell growth phase also prevented any glucose limitation and the excessive accumulation of lactate (Fig. 1c).

As an alternative to the Hybrid 1, the Hybrid 2 was started at 8 × 10^6^ cells/mL and cultivated up to 26 × 10^6^ cells/mL. The resulting CSPR and *μ*_mean_ were in average 0.057 nL/(cell × day) and 0.024 1/h, respectively (Table 1). These were also in accordance with the target values of 0.06 nL/(cell × day) and 0.026 1/h. In contrast to the Hybrid 1 cultivation, the cell suspension was concentrated twofold before infection by reducing the working volume to 0.3 L (Fig. 1d), and one reactor volume was exchanged with fresh medium. This procedure shortened the cell growth phase to 2 days and obviated discarding half of the volume of the produced cell suspension (Fig. 1e). Afterwards, the already described hybrid strategy was applied. The on-line VCV monitoring implemented in Hybrid 2 correlated well with off-line measurements up to late stages of the MVA virus propagation phase (Fig. 1e). This demonstrated the robustness of on-line capacitance measurements when the VCD exceeds 40 × 10^6^ cells/mL, and expanded measurement to stages where virus-induced cell damage and apoptosis are widely spread within the infected cell population. In general, both the manually controlled CSPR during the cell growth and the hybrid strategy applied during virus propagation prevented the glucose limitation and the excessive accumulation of lactate (Fig. 1f).

**Fig. 2:**
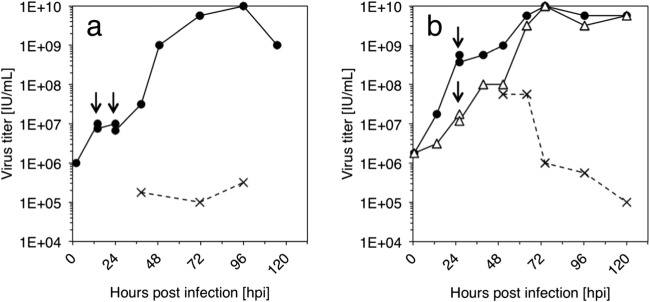
Progression of the MVA-CR19 virus production for two hybrid FB/perfusion variants, Hybrid 1 (**a**) and Hybrid 2 (**b**). Virus titers of whole cell lysates (filled circles) and permeate (crosses) are indicated for both variants. Virus titers in the supernatant (triangles) were determined only for Hybrid 2 (**b**). Arrows: time points of bolus feeding

A maximum MVA-CR19 virus yield of 1.0 × 10^10^ IU/mL was obtained at 96 hpi and 72 hpi for Hybrid 1 and Hybrid 2, respectively (Fig. 2). For Hybrid 1, the virus particles were harvested using the same ATF hollow fiber module (0.65 μm pore size) as in the cell growth phase, whereas for Hybrid 2 a 0.2-μm module was used during the cell growth phase and replaced by a new 0.65-μm module for perfusion and virus harvest at 36 hpi. The relatively low virus harvest observed in Hybrid 1 (Fig. 2a) suggested that membrane fouling might have occurred during the cell growth phase. Nevertheless, a similar maximum virus titer, cell-specific yield, and volumetric productivity were obtained compared to the reference process in shake flasks (Table 2, F + D), which involved daily harvesting of virus (Vazquez-Ramirez et al. 2018). Even more significant is the more than tenfold increase in both the cell-specific yield and the volumetric productivity of Hybrid 1 compared to the use of only perfusion during virus propagation (Table 2, Perfusion) (Vazquez-Ramirez et al. 2018). Given the suboptimal virus harvesting for Hybrid 1, the product collected in the permeate was not considered for the calculation of the cell-specific yield and the volumetric productivity.

For Hybrid 2, almost all infectious virions were found in the culture supernatant with a maximum virus titer of 1 × 10^10^ IU/mL (72 hpi, Fig. 2b). This is in agreement with previous reports (Jordan et al. 2013; Vazquez-Ramirez et al. 2018). Changing the hollow fiber module during the virus production phase enabled a quantitative harvest of virus particles from the supernatant only during the first 12 h of perfusion (i.e., 36–48 hpi) (Fig. 2b). From 48 hpi, the virus particle concentration in the permeate decreased considerably with respect to the apparent virus content in the culture supernatant. The virus titer in the permeate reached 1 × 10^6^ IU/mL at 72 hpi, when the maximum virus titer of 1 × 10^10^ IU/mL was reached in the culture supernatant (Fig. 2b). A further decrease in the permeate virus concentration to 1 × 10^5^ IU/mL was observed up to 120 hpi, while the titer in the bioreactor remained almost stable in the order of 10^9^ IU/mL (Fig. 2b). Similar to the Hybrid 1 cultivation, these observations suggest significant membrane fouling at 48 hpi (24 h after begin of perfusion), preceding the most productive period of the virus propagation phase that occurs 48–72 hpi (Fig. 2b). This phase also coincides with a fast drop of cell viability and, very likely, with increasing in cell lysis. The resulting massive accumulation of cell debris and the aggregation of MVA-CR19 viral particles (200–400 nm) may eventually have led to the obstruction of the ATF membrane (0.65 μm nominal pore size). Maximum titers were reached 72 hpi, and infectious virions appeared to remain stable in the supernatants and as well in the total cell lysates, as no obvious decay in titers was observed up to 120 hpi (Fig. 2b).

Despite the differences in the feeding profile during the FB, the Hybrid 2 variant was as productive as Hybrid 1 (Table 2). In particular, a cell-specific virus yield of 352 IU/cell and a volumetric productivity of 2.8 × 10^11^ IU/(L × day) were obtained. Similar to the Hybrid 1 variant, a very low amount of product was collected in the permeate line and, therefore, was neglected for the calculations of the cell-specific yield and the volumetric productivity. The reduced process time of Hybrid 2 (8.2 days) led to a twofold higher volumetric productivity compared to cultivation Hybrid 1. However, virus yields in the order of 10^11^ IU/(L × day) would still be expected for a process operated at a similar time period as cultivation Hybrid 1 (10.9 days). As the cell-specific virus yields were comparable to Hybrid 1 (Table 2), the use of both strategies seems reasonable.

### Influenza A virus propagation using hybrid FB/perfusion

An earlier study that investigated factors interfering with the production of influenza A virus at HCD gave only partial solutions regarding productivity optimization (Genzel et al. 2014). Perfusion bioreactors were optimized in this earlier study with the avian cell line AGE1.CR in such a way that the cell-specific virus yields increased. However, overall volumetric productivity of these processes was higher in batch cultivations performed at conventional cell densities. The AGE1.CR cell line is the parental cell line of CR.pIX cells and has shown higher productivities for influenza A virus. In contrast, the productivity was slightly lower for MVA virus compared to CR.pIX cells (Jordan et al. 2009; Lohr et al. 2009).

**Fig. 3:**
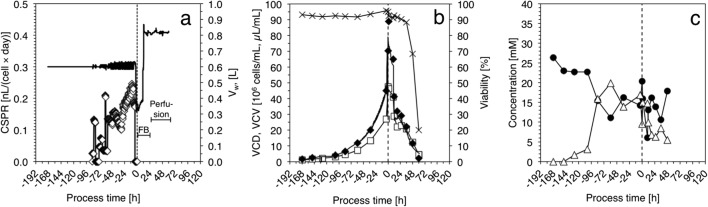
Cultivation parameters and cell growth for a hybrid FB/perfusion process for the poduction of influenza A/PR/8/34 (H1N1) virus at high cell density. **a** Cell-specific perfusion rate, CSPR, (empty diamonds) and working volume, *V*_w_ (continuous line). **b** Viable cell density, VCD (squares); on-line viable cell volume, VCV (continuous line); off-line VCV (full diamonds) (for VCV calculations see “Materials and Methods” section), and viability (crosses). **c** Glucose (filled circles) and lactate (triangles) concentration. Time of infection, 0 h (vertical dashed line)

Accordingly, it was investigated next whether the hybrid FB/perfusion strategy established for MVA virus in this study could also be used for improving the volumetric productivity of influenza A/PR/8/34 (H1N1) virus in CR.pIX cells at HCD. In addition, an alternative perfusion control was evaluated for the cell growth phase based on the glucose consumption, lactate accumulation, and the resulting medium acidification. To accomplish this, harvest and medium feeding pumps were activated when pH dropped to values below 7.2 and turned off when the pH increased to 7.2. Under this condition, glucose is replenished at the same time that lactate is removed from the bioreactor. This perfusion control allowed for an average CSPR of 0.119 nL/(cell × day) (Table 1, Fig. 3a) and cell viabilities above 92% (Fig. 3b). Additionally, it enabled to maintain glucose concentrations above 10 mM (Fig. 3c), similar to a previous report in shake flasks, where the pH was controlled in a range of 7.2 ± 0.2 when applying a CSPR of 0.06 L/(cell × day) with fresh CD-U3 medium (Vazquez-Ramirez et al. 2018). Different to the HIPCOP strategy proposed by Hiller et al. (2017), which operates at glucose limitation and a lactate consumption regime in CHO cell cultivations, this strategy allowed for a perfusion control without reaching low glucose concentrations that might negatively affect the growth of CR.pIX cells.

After the CR.pIX cells were cultivated to 27 × 10^6^ cells/mL, the *V*_w_ was reduced from 0.6 to 0.3 L and 1 reactor volume was exchanged with fresh medium before virus infection (Fig. 3a). A final concentration of 47 × 10^6^ cells/mL was measured just before infection (Fig. 3b). Influenza A viruses are reported to replicate rapidly in CR.pIX cells (Jordan et al. 2016; Lohr et al. 2009). An increase in HA titers is typically observed at about 6 hpi and, depending on the virus strain, maximum titers are obtained 24–36 hpi (Lohr et al. 2009). Based on this information, and in contrast to the production of MVA virus, the FB phase was shortened (0–12 hpi), followed by a perfusion phase at a rate of one reactor volume per day (Fig. 3a). A process operated completely in perfusion and with total virus retention, as proposed by Genzel et al. (2014), was performed as a reference process (Online Resource 1).

**Fig. 4:**
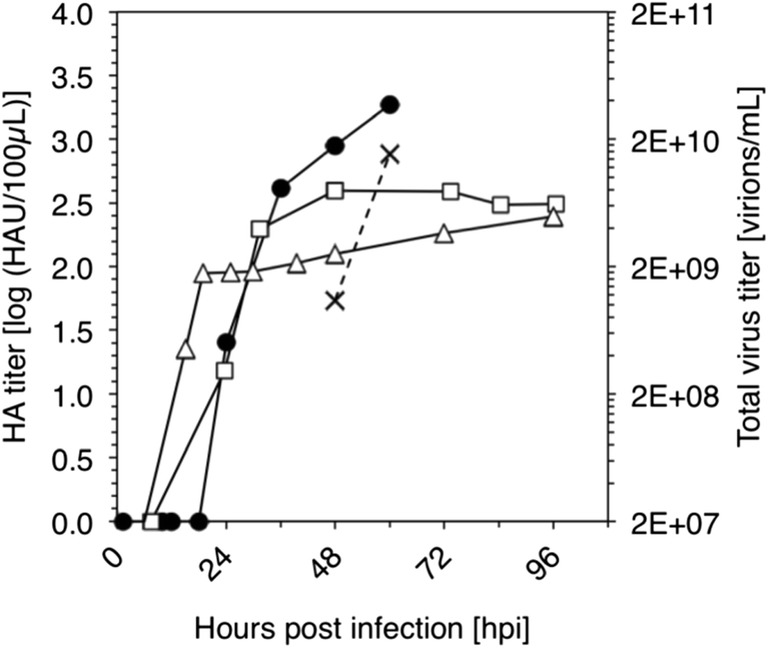
Progression of influenza A/PR/8/34 (H1N1) virus production for different cultivation strategies. HA titers and the corresponding total number of virions/mL are indicated for hybrid FB/perfusion (bioreactor supernatant: filled circles, permeate: crosses), perfusion (squares), and batch (triangles)

The hybrid cultivation yielded a maximum HA titer of 3.3 log HAU/100 μL (3.8 × 10^10^ virions/mL) at 60 hpi (Fig. 4). This represented a fivefold increase compared to the reference perfusion cultivation (Fig. 4). Compared to a conventional batch process (Table 2), the increase was sevenfold.

## Discussion

The implementation of the hybrid strategy in benchtop bioreactors showed a similar performance compared to analogous experiments in shake flasks and led to a clear improvement of virus yields towards perfusion (for HCD) and batch (for conventional cell densities). Next, broader benefits in relation to reported production strategies with different cell substrates and its advantages for a potential industrial application were analyzed.

### MVA-CR19 virus propagation using hybrid FB/perfusion

Although CD-U3 medium was not developed for perfusion processes (Jordan et al. 2011), medium consumption to achieve concentrations of about 50 × 10^6^ cells/mL with CSPR-based perfusion was moderate (6.15 reactor volumes for Hybrid 1 and 1.98 reactor volumes for Hybrid 2). Despite the low medium utilization, the average cell-specific growth rates for both Hybrid 1 and 2 cultivations were comparable to the 0.023 1/h achieved previously in shake flasks (Vazquez-Ramirez et al. 2018). Accordingly, it also took only about 8 days to reach a minimum VCD of 50 × 10^6^ cells/mL. Petiot et al. (2011) reported a medium utilization of about 3.5 reactor volumes to expand HEK293 cells within 9 days from 0.25 to 15 × 10^6^ cells/mL (before infection with influenza virus). Genzel et al. (2014) reported a medium consumption of 11.3 reactor volumes to propagate AGE1.CR cells to 50 × 10^6^ cells/mL before infection with influenza virus. Therefore, the results here represent a significant reduction in medium consumption before virus infection, which is an important contribution towards lower COGs in large-scale production.

Both Hybrid 1 and Hybrid 2 variants simplify the production process because a single bioreactor can be used for cell expansion and virus propagation. In Hybrid 1 cultivation, one-half of the cell suspension (0.3 L) was removed before infection at 50 × 10^6^ cells/mL (Fig. 1 a).This cell suspension could possibly be used to start a second bioreactor in parallel. In contrast, in the Hybrid 2 cultivation, cells were cultivated to 25 × 10^6^ cells/mL in 0.6 L and concentrated to 50 × 10^6^ cells/mL prior to infection (Fig. 1 d). In both cases, the subsequent FB phase required the addition of almost three times the starting volume to avoid substrate limitations. This ratio was lower than the 1:4 reported by Pohlscheidt et al. (2008) for the high-yield production of *Parapoxvirus ovis* at large scale, which—in addition—required transferring the cell suspension to a second larger bioreactor to perform the dilution steps. Since the initial FB phase of the hybrid strategy seems to be a critical operation also for MVA-CR19 virus propagation (Vazquez-Ramirez et al. 2018), further studies could focus on the development of an optimized feed medium to enable a higher starting volume (preferably 60% of the maximum working volume) and a lower maximum dilution ratio (about 2:3) to simplify the hybrid strategy for implementation in large-scale bioreactors.

Overall, the established hybrid strategies for MVA-CR19 virus production (Table 2, Hybrid 1 and Hybrid 2) resulted in a 10 to 100-fold increase in virus titers compared to the current standard production platform in CEF cells (Gilbert et al. 2005; Meiser et al. 2003). With respect to cultivations performed at conventional cell densities using CR.pIX cells (Jordan et al. 2009; Lohr et al. 2009; Lohr 2014), EB14 cells (Guehenneux and Pain 2005), and EB66 cells (Léon et al. 2016), up to tenfold higher titers were obtained. Cell-specific virus yields obtained with the hybrid strategies (410 and 352 IU/cell) were also competitive regarding the 500 IU/cell obtained with CEF cells (Carroll and Moss 1997), the 50–200 IU/cell with CR.pIX cells (Lohr 2014), and the 25–50 IU/cell with EB66 cells (Léon et al. 2016) at conventional lower cell densities. Batch production of MVA virus with CR.pIX cells (Jordan et al. 2009; Lohr 2014) and EB66 cells (Léon et al. 2016) requires more or less the same time and the same media volumes. Accordingly, its volumetric productivity of about 2.0 × 10^10^ IU/(L × day) is clearly surpassed by the 1.3 and 2.8 × 10^11^ IU/(L × day) obtained with the hybrid strategy. Applying such a strategy would allow for 100,000 doses per liter of cell-free supernatant, considering that single doses of 1 × 10^8^ PFU (1 PFU = 1 IU) per individual are currently used in clinical studies involving recombinant MVA-based vaccines (Gomez et al. 2013).

Due to its application as a viral vector, maintaining the infectious activity of MVA is a critical quality attribute. It is promising that titers were found to remain stable from 72 to 120 hpi (Fig. 2b). This suggests a low virus inactivation rate for the specific cultivation conditions chosen. While continuous virus harvesting failed for the chosen ATF system, the use of other cell retention devices including acoustic filter and settlers might be evaluated again for large-scale production to avoid product losses. One major property of the MVA-CR19 virus, its capacity to propagate in true single-cell suspension cultures, may additionally help to facilitate the recovery of infectious units directly from the culture supernatant without the need of cell disruption (Jordan et al. 2013). The Hybrid 2 cultivation showed that the maximum titer at 72 hpi accounted entirely for virus in the supernatant (Fig. 2b) with most of the cells showing a viability > 70%. Hence, a clarification step at this point with a carefully chosen cell retention system would suffice to recover the MVA-CR19 virus from the bioreactor. Based on the very high performance of the hybrid strategy in upstream processing and further options to reduce costs in downstream processing, it can be assumed that higher costs related to the implementation of “complex” perfusion processes (purchase of dedicated equipment and training of staff) can be more than compensated even at industrial scale.

### Influenza A virus propagation using hybrid FB/perfusion

The perfusion control applied during the cell growth phase in the hybrid cultivation led to a *μ*_max_ = 0.025 1/h and an overall *μ*_mean_ = 0.019, which were in accordance with previous HCD bioreactor cultivations (Table 1). Similar to the Hybrid 2 process for MVA-CR19 virus, on-line VCV estimations correlated well with off-line measurements up to late stages of the influenza A virus propagation phase (Fig. 3b). No glucose limitation nor significant lactate accumulation was observed for the pH-based perfusion control during the cell growth phase (Fig. 3c). The high average CSPR of 0.119 nL/(cell × day) (Table 1, Fig. 3a) obtained in the cell growth phase led to an increase in medium consumption (16.7 reactor volumes) compared to the reference perfusion process (3.9 reactor volumes). Since the perfusion rates depended on the pH control of the cultivation, reducing the pH set point could further minimize the high medium exchange.

Despite the very high medium consumption, the hybrid strategy provided a cell-specific yield of 1300 virions/cell and a volumetric productivity of 5.4 × 10^11^ virions/(L × day) (Table 2). This observation confirms that the cell density effect can be circumvented by a hybrid strategy for influenza A virus-infected cultures. The difference of 17% in volumetric productivity, with respect to the batch cultivation, can be explained by the rather high medium consumption during the cell growth phase of the hybrid cultivation. Compared to the perfusion-only strategy, the hybrid FB/perfusion conferred a clear improvement in both cell-specific and volumetric productivity (Table 2), and showed comparable yields for influenza A virus with respect to the parental suspension cell line AGE1.CR (Genzel et al. 2014).

Process intensification by consequent use of HCD strategies in viral vaccine manufacturing can contribute to a stable supply of vaccines. In case conventional batch production processes are already established, the implementation of HCD strategies can significantly increase manufacturing capacities, e.g., in emerging and developing countries where vaccines are most urgently needed. A possible solution using a hybrid FB/perfusion strategy during the virus production phase for MVA and influenza A virus in small stirred tank bioreactors is described here. The application of this strategy resulted in a 7- to 20-fold increase in virus titers without compromising cell-specific yields and volumetric productivities that often hinder the establishment of intensified processes. The high titers of 10^10^ IU/mL obtained for MVA virus demonstrated, in particular, the potential of this approach as an alternative to the current technology that relies on primary chicken embryo fibroblasts as a substrate. The results achieved here for the two different viruses may be also instructive for modernization of conventional approaches in viral vaccine production.

## Electronic supplementary material

ESM 1 (PDF 223 kb)
