# Supplementary material for: High titer MVA and influenza A virus production using a hybrid fed-batch/perfusion strategy with an ATF system
Source: Appl Microbiol Biotechnol. 2019 Feb 23;103(7):3025–35. doi: 10.1007/s00253-019-09694-2 (PMC6447503; doi:10.1007/s00253-019-09694-2)
Supplement: Supplementary file 1 — (PDF 223 kb) [file 253_2019_9694_MOESM1_ESM.pdf]

Applied Microbiology and Biotechnology

High titer MVA and influenza A virus production using a hybrid fed-batch/perfusion strategy with an ATF system

Daniel Vázquez-Ramírez[1], Ingo Jordan[2], Volker Sandig[2], Yvonne Genzel[1*], Udo Reichl[1,3]

[1]Max Planck Institute for Dynamics of Complex Technical Systems, Sandtorstr. 1, 39106, Magdeburg, Germany

[2]ProBioGen AG, Goethestr. 54, 13086 Berlin, Germany

[3]Chair for Bioprocess Engineering, Otto-von-Guericke-University Magdeburg, Universitätsplatz 2, 39106 Magdeburg, Germany

*Corresponding author

Email address: genzel@mpi-magdeburg.mpg.de

Keywords: Viral vaccine production - Process intensification - On-line monitoring

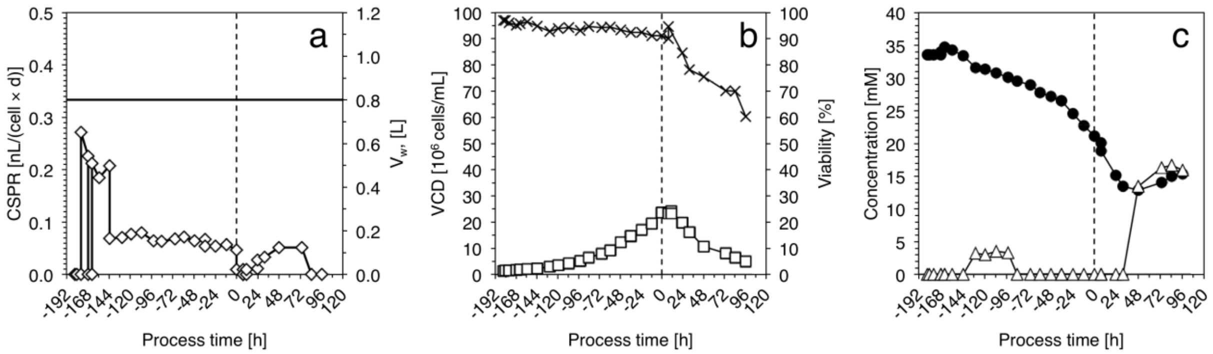

Fig. S1 Reference perfusion process for the production of influenza A/PR/8/34 (H1N1) virus at 24 × 10^6 cells/mL. Perfusion was started 6.75 h after cell inoculation with a cell-specific perfusion rate of 0.06 nL/(cell × day). a: cell-specific perfusion rate, CSPR, (empty diamonds) and working volume, V_w (continuous line). b: viable cell density, VCD (squares) and viability (crosses). c: glucose (filled circles) and lactate (triangles) concentration. Time of infection: 0 h (vertical dashed line)
